# Research on the health status and influencing factors of the older adult floating population in Shanghai

**DOI:** 10.3389/fpubh.2024.1361015

**Published:** 2024-05-22

**Authors:** Lianxia Wu, Wei Li, Shaogu Wang, Guan Weihua, Xianyu Wang

**Affiliations:** ^1^Population Research Institute, School of Social Development, East China Normal University, Shanghai, China; ^2^School of Geography and Planning, Sun Yat-sen University, Guangzhou, China; ^3^School of Geography, Nanjing Normal University, Nanjing, China; ^4^Collaborative Innovation Center for Development and Utilization of Geographic Information Resources in Jiangsu Province, Nanjing, China

**Keywords:** older adult floating population, multidimensional characteristics, health status, influencing factors, Shanghai

## Abstract

**Introduction:**

Over the past decade, against the dual background of population aging and mobility, the older adult/adults floating population has become a new type of mobile group in China, continually congregating in large cities, posing significant challenges to the socio-economic development, eldercare services, and public management of these metropolises. Shanghai, as a mega-city and the economic center of the China, is typically representative of the national population.

**Methods:**

Based on the dynamic monitoring data of Shanghai’s floating population in 2018, this research uses mathematical statistics and binary Logistic regression models.

**Objective:**

This research analyzes the demographic characteristics and health status of the older adult/adults floating population in Shanghai in the new era and reveals its primary influencing factors.

**Results and discussion:**

(1) A prominent contradiction in the scale and structure of the older adult/adults floating population, with widowed and low-educated mobile older adult/adults requiring attention. (2) There is a lack of health knowledge, and the proportion of local reimbursement is low. Over 90% of migrant older adult/adults self-assessed their health (with a very few unable to care for themselves), far higher than the proportion of older adult/adults who are not sick (injured) or uncomfortable (actually healthy), which exceeds 70%. The health status of migrant older adult/adults deteriorates with age, and those who have never attended school and live alone have the worst health status. (3) Older adult/adults people with advanced age and low educational levels are at risk of health issues, while a better living environment can reduce the risk of illness in the older adult/adults floating population. Low family income, poor housing affordability, and the medical burden brought about by illness can easily lead to older adult/adults floating populations falling into the trap of older adult/adults poverty, and older adult/adults people from central regions and those who migrate along have difficulty adapting to city life, leading to poor self-assessed health. Meanwhile, community/enterprise health education helps to enhance the health protection awareness of the older adult/adults floating population. Finally, based on the governance concept of “mobility publicness,” several public management and service optimization strategies for social support for the older adult/adults floating population in Shanghai are proposed.

## 1 Introduction

In the context of demographic transition, the older adult floating population has become a new type of mobile group in China. On the one hand, with the extension of life expectancy, people’s mobility increases after retirement, making old age a new peak of mobility after a continuous decline in the rate of migration since middle age. On the other hand, with the deepening degree of population aging, the proportion of the older adults in the migrant population is also increasing correspondingly (1). According to the seventh National Census, as of 2020, the older adult population aged 60 and above in China accounted for 18.7% of the total population; the migrant population was as high as 376 million, accounting for 26.6% of the total population. National Bureau of Statistics data shows that in 2021, China’s migrant population reached 385 million, while the total population aged 60 and above exceeded 260 million. It is evident that under the dual massive scales of the aging population and the migrant population, the scale of the older adult floating population is rapidly increasing (2).

The older adult floating population, with the dual characteristics of “aging” and “mobility,” faces health risks that urgently require the attention of the government and academia. “Aging” signifies a gradual degradation of physiological functions, while “mobility” may bring about issues of environmental adaptation and policy neglect, posing considerable challenges to social governance. Compared to other population groups, the health issues of the older adult floating population have not received sufficient attention and research, and policy concern for the older adult floating population and their health status is relatively inadequate.

Shanghai, as the economic center of the country and a mega-city, is typically representative and significant on the issue of the older adult floating population. A large number of older adult floating population are pouring into Shanghai, putting tremendous pressure on Shanghai’s socio-economic development, public services, and eldercare services. The health status of the older adult floating population not only directly affects their quality of life but is also closely related to Shanghai’s rational layout of eldercare service facilities, the implementation of social eldercare policies, and the promotion of equal public services. The report from the 20th Party Congress points out: “Promote the construction of a healthy China. Put the guarantee of people’s health in a strategic position of priority development, and improve the policy of promoting people’s health.” Therefore, in-depth research on the health status of the older adult floating population in Shanghai and its influencing factors is beneficial to strengthen policy attention and provide more equitable health services. It is of significant practical significance to provide experiences and references for other cities, improve the health status of the older adult floating population, promote equal public services, and implement healthy aging policies.

The academic community has conducted comprehensive research on the health status of the older adult floating population and the factors that influence it. Current research on the health status of the older adult floating population primarily uses self-assessed health, chronic disease, and daily living self-care abilities to measure health levels, with some researchers focusing on mental and social health (3, 4). Among these, the self-assessed health indicator not only reflects the comprehensive status of subjective and objective, past and present health, but also includes future health status, resistance to disease, and the degree of concern about health (5). Some researchers believe that self-assessed health is even more important than actual medical measurement results (6). Chronic disease indicators can objectively reflect physiological health status. Mental health is generally measured by anxiety or depression scales, and social health refers to the interaction between individuals and others, as well as social systems and customs, and the adaptation to social life (7). Existing research shows that the overall health status of China’s current older adult floating population is good, but it is influenced by various factors, and there is still internal differentiation and potential health risks (8–12). The factors influencing the health status of the older adult floating population can be divided into six categories: demographic factors, socio-economic factors, health and medical factors, lifestyle factors, migration factors, and social support factors. In terms of demographic factors, a representative view is that older adult floating populations who are male, younger, and have a stable spouse usually have better health status. Compared with the older adult floating population with a higher level of education, those with a lower level of education have a lower level of self-assessed health and a higher incidence of chronic diseases (13–16). Some research also points out that the health status of middle-aged migrant older adults (70–79 years old) is worse than that of the younger migrant older adults (60–69 years old), and urban household registration migrant older adults can control more health resources, which can buffer the impact of education on health status to a certain extent (17, 18). In terms of socio-economic factors, the health status of older adult floating populations with lower income levels or whose main source of income is family support is usually worse. Health and medical factors are mainly divided into three categories: health education or communication, medical habits, and access to medical resources. A representative view is that health communication significantly promotes the improvement of the health status of migrant older adults through mechanisms such as medical service accessibility, medical service utilization, and health literacy (19). Public health education can also improve the self-assessed health level of migrant older adults, and its impact on the health of rural migrant older adults in the eastern region with high age and high cultural level is greater (20–22). Participating in health check-ups and seeking medical attention for minor illnesses has a positive effect on the health status of migrant older adults. In terms of access to medical resources, the self-assessed health level of migrant older adults who are close to medical places and have established health files is lower (23). Compared with uninsured older adult floating populations, insured older adult floating populations have a higher confirmed rate of chronic diseases. Social health insurance can improve the health level of the older adults. However, some research also shows that social health insurance focuses on economic compensation and has a lag in health protection, and it does not have a significant positive effect on the health of migrant older adults (24). Although insurance has not improved the health status of the older adults or reduced the mortality rate of the older adults, it can significantly increase the utilization rate of outpatient, inpatient, and preventive health care services for the older adults (25, 26). It is worth noting that the probability of migrant older adults using health services is low (27), and there is inequality in the acquisition of basic public health services in terms of identity, region, and city, and the acquisition of basic public health services is at a disadvantage compared to local older adults, and there are differences in urban and rural household registration and differences in the east, middle, and west regions (28, 29). In terms of lifestyle factors, migrant older adults who do not have smoking and drinking habits, exercise for a long time daily, and participate in social activities usually have a higher level of self-assessed health. Quality sleep, longer residence time, and intergenerational consensus have a positive effect on the mental health of the migrant older adult population. In terms of migration factors, a representative view is that older adults with poor health status have characteristics such as long-term mobility, multi-city mobility, mobility with companions, migration for the purpose of caring for family members and older adult care, migrating to remote areas in the northeast or northwest, the size of the city where they migrated to is reduced and the planning direction does not conform to the “dense road network” (emphasize the connectivity, accessibility, and reliability of the road system), and the communication and leisure environment of the community where they live is poor (16–18, 30). Contrary to the conclusions drawn from previous studies, it has been found that the health conditions of the migrant population improve over a longer period of time. This could be due to their adaptation to the culture in the later stages of migration and gaining access to abundant social resources (31). Some research also found that older adults who migrate for the reason of taking care of their descendants have better self-assessed health than those who work and do business, and the self-assessed health level of rural household registration migrant older adults decreases with the increase of migration time, but this effect is not significant for urban household registration migrant older adults. Some studies also point out that population migration affects the mental health of migrant older adults through two opposite mechanisms: health selection and migration risk. In terms of social support factors, the social support network of the older adult floating population shows characteristics of small scale, high closeness, low heterogeneity, and high homogeneity, and the social support network has a direct positive effect on the health of the older adult floating population (8, 15). Specifically, the spousal support, family economic support, and the number of friends in the place of migration have a significant positive impact on the self-assessed health of the older adult floating population, and social interaction and community service levels are important factors affecting the mental health status of migrant older adults (32–34).

At the theoretical level, in view of the characteristics of China’s older adult floating population, existing research has combined Western migration theory and general population health-related theory to lay the following foundational understanding for explaining the health status of older adult floating population and predicting future trends. First, as the most important foundational theory in the study of the health of China’s older adult floating population, the Healthy Migrant Theory emphasizes the good physical and mental health conditions required for migration, that is, migrants are healthier than those who have not migrated from their original places of residence. Second, Cultural Function Theory and Transnationalism focus on the health of migrants being jointly affected by the cultural forces of both the place of immigration and the place of emigration, which may be realized through social relationships, social support, etc. Third, Social Capital Theory highlights the negative impact of social exclusion and isolation on the health of migrants, and the concept of structural vulnerability also analyzes how administrative irregularities and policies targeting immigrants create unfair socio-political-economic environments that in turn affect immigrant health. Lastly, SDOH (social determinants of health) discusses a variety of processes that may have negative impacts on migrant health, such as overcrowding, environmental health, and multi-level poverty, and also emphasizes the relevance of health with social environment and social strata (35, 36).

Existing research has explored the health status of the older adult floating population and influencing factors, but there are still shortcomings. First, the concept of the older adult floating population is relatively vague. Current research lacks a consistent understanding of age, migration time, and administrative regions crossed by migration, making the conclusions of different studies less comparable (37). Second, existing research shows that the internal heterogeneity of the older adult floating population is high, so it is necessary to group them socially when studying their health, but some research has not grasped this characteristic. In response to the aforementioned shortcomings, this research, based on existing research, believes that the older adult floating population refers to the population aged 60 and above who have left their place of household registration (county-level or above) for 1 month or more without changing the nature of their household registration. Besides, the older adult floating population can be grouped from multiple perspectives to analyze their heterogeneity in this paper, such as dividing them into 5 groups (60–64 years old, 65–69 years old, 70–74 years old, 75–79 years old, and 80 years old and above) from an age perspective. In summary, this study comprehensively analyzes the characteristics and health status of the older adult migrant population in Shanghai in the new era, revealing the main factors affecting the health of the older adult migrant population in Shanghai. It provides empirical evidence for the next step in advancing the policy formulation for the older adult migrant population. The research has significant practical implications for enhancing the attention to the policies of the older adult migrant population, promoting the equalization of public and health services, and responding to the implementation of the proactive strategy to cope with aging at the national level.

## 2 Methods, variables and data sources

The study utilized a binomial Logistic regression model in the SPSS software to analyze multiple factors affecting the health status of the older adult migrant population in Shanghai. The model formula is as follow (38):


(1) $$\mathrm{Logit}\left(p\right)=\ln\frac{p}{1-p}$$

(2) $$\ln\frac{P\left(Y=1|X\right)}{1-P\left(Y=1|X\right)}=\beta_{0}+\beta_{1}X_{1}+\beta_{2}X_{2}+\cdots +\beta_{n}X_{n}$$

In this equation, *P(Y = 1|X)* represents the conditional probability of the dependent variable under the given independent variable *X. β_0_*, *β_1_…β_n_* are the parameters of the model, where *β_0_* is the intercept and *β_1_* to *βn* are the weights of each independent variable, indicating the degree of influence each independent variable has on the outcome.

Empirical analysis in this paper uses the dynamic monitoring data of the migrant population in Shanghai in 2018 from the “China Migrants Dynamic Survey.” The CMDS adopts a hierarchical, multi-stage, and scale-proportional PPS sampling method, taking the non-local population aged 15 and above who have resided locally for more than 1 month as the survey objects. It conducts a detailed investigation of the basic individual and family characteristics of the floating population, as well as the basic conditions of the inflow and outflow areas. The survey covers 31 provinces (regions, cities) and the Xinjiang Production and Construction Corps.

This Research selects the older adult floating population aged 60 and above as the main research objects, with a total of 361 valid samples after screening. To simplify the results of data analysis, besides the overall description, the four options in health self-assessment are grouped into “healthy” (combining “healthy” and “basically healthy”) and “unhealthy” (combining “unhealthy, but can take care of oneself” and “unable to take care of oneself”). The questionnaire on the condition of the older adult floating population falling ill (injured) or feeling unwell in the past year is also simplified, combining “fell ill within 2 weeks” and “fell ill more than 2 weeks ago” into “fell ill,” and changing “no” to “not ill” (the same rule applies to the related content below). That is to say, in the CMDS survey questionnaire, for the question “Have you ever been sick (injured) or feeling unwell in the past year?,” the answers “1 Yes, the last occurrence occurred within 2 weeks” and “2 Yes, the last occurrence occurred 2 weeks ago” are classified as “fell ill “in this article, while the answer “3 No” is considered “not ill.” “Self- assessed health” and “actual disease status” are both binary variables, with self-assessed as “healthy” = 1 and “not ill” =1 (see from Table 1). The two outcome variables of health self-assessment and illness status reflect both subjective and objective aspects of the health status of the older adult floating population and are binary variables in this article (5, 7).

**Table 1:** Description of variables.

Types of variables		Variable name and variable description
Outcome variables	Health status	Self-assessed health (self-assessed as “healthy” = 1)
		Disease status (not sick = 1)
Explanatory variables	Individual factors	Gender (male = 1)
		Ages (60–64 years old = 0)
		Education level (never attended school = 0)
		Marital status (without spouse = 0)
	Socio-economic factors	Household registration type (agricultural = 0)
		Household monthly income (low-income group = 0)
		Housing expenditure (low = 0)
	Migration factors	Place of household registration (East = 0)
		Length of stay in Shanghai (0-4 years = 0)
		Reason for migration (working/doing business = 0)
		Willingness to settle (No = 0)
	Healthcare factors	Health education (No = 0)
		Medical insurance (No = 0)

Incorporating existing research, theories of social determinants of health (SDOH) and data availability, this study selects individual factors, socio-economic factors, health care factors, and migration factors as explanatory variables (8–25, 35). Shown in Table 1, individual factors include age, gender, education level, and marital status (with or without a spouse); socio-economic factors include household registration type, housing expenditure (divided into high, medium, and low groups, with the low-expenditure group as the reference group), and family monthly income (divided into three types: low income group of 694$ and below, medium income group of 695–2083$, and high income group of 2084$ and above, with the low income group as the reference group). Health care factors include whether to receive community/unit health education and whether to participate in insurance; migration factors include the length of stay in Shanghai (divided into 0–4 years, 5–9 years, 10–14 years, 15–19 years, and 20 years and above, with 0–4 years as the reference group), reasons for migration (divided into working/trading, family migration, visiting friends and relatives, and living in a different place for old age, with working/trading as the reference group), whether there is a willingness to stay, and the province of household registration (divided into east, central, west, and northeast, with east as the reference group). It should be noted that the indicator of the province of household registration reflects the inflow area of the older adult floating population. Existing research has found that the health status of the older adult floating population migrating to remote areas in the northeast or northwest is relatively poor, and because the level of economic development and medical social security in the eastern region is more advanced in China, the eastern region is set as the reference group (17).

## 3 Descriptive results of health status and medical security of older adult floating population

### 3.1 Descriptive results of health and disease status of older adult floating population

#### 3.1.1 Self-assessed health and disease status

From the overall self-assessment results, the health status of Shanghai’s older adult floating population is good. As Table 2 shown, the proportion of older adult floating population in Shanghai who self-assessed as “healthy” was 54.1%, and the proportion of those who self-assessed as “basically healthy” was 37.4%, together accounting for 91.5%. Among the older adult floating population who self-assessed as “unhealthy,” only 0.7% were unable to take care of themselves, and the majority of older adult floating population, although unhealthy, still have the ability to live independently (To more thoroughly exhibit the distribution of the research subject’s self-assessment of health and illness conditions, this section employs the classification types from the original questionnaire).

**Table 2:** Self-assessed health status.

Self-assessed health status	Healthy	Basically healthy	Unhealthy, but able to take care of oneself	Unable to take care of oneself	Total
Proportion (%)	54.1	37.4	7.8	0.7	100.0

Compared with the self-assessment results, the proportion of older adult floating population in Shanghai who are ill (injured) or physically uncomfortable is relatively low. As Table 3 shown, the proportions of older adult floating population who became ill (injured) or physically uncomfortable within the last 2 weeks and those before that were 9.6 and 12.3%, respectively, while the proportion of older adult floating population who did not become ill (injured) or physically uncomfortable was 78.1%, far lower than the total of those who self-assessed as healthy and basically healthy (91.5%). This suggests that older adult floating population in Shanghai may have overestimated their health status in self-assessment. This may be related to the lack of medical knowledge among the older adult floating population. They are unclear whether the condition of being ill (injured) or physically uncomfortable falls within the scope of health, and mistakenly understand some chronic diseases in the older adults as normal phenomena, resulting in a self-assessed health status that is much higher than the actual non-illness rate.

**Table 3:** Illness (injury) or physical discomfort.

Illness (injury) or physical discomfort	Sick within 2 weeks	Sick more than 2 weeks ago	No	Total
Proportion (%)	9.6	12.3	78.1	100.0

#### 3.1.2 Self-assessed health status and individual factors

##### 3.1.2.1 Men’s self-assessed health is polarized, women are in the middle, but men’s actual non-illness rate is higher than women’s

Overall, the proportions of men and women among the older adult floating population in Shanghai who self-assessed as healthy are the same, and there is no significant gender difference yet (Table 4). However, a detailed analysis reveals that the proportion of men who self-assessed as “healthy” (58.3%) is higher than that of women (50.5%), the proportion of women who self-assessed as “basically healthy” (41.1%) is higher than that of men (33.3%), the proportion of women who self-assessed as “unhealthy, but able to live independently” (8.3%) is higher than that of men (7.1%), and all those who “cannot live independently” are men (1.2%), with no women. It can be seen that men’s self-assessed health status is polarized, with the proportions of “healthy” and “unable to live independently” both higher than women’s, while women’s self-assessed health status is relatively in the middle, mainly “basically healthy.”

**Table 4:** Health self-assessment status by gender.

Health status	The proportion of male (%)	The proportion of female (%)
Healthy	58.3	50.5
Basically healthy	33.3	41.1
Unhealthy	8.3	8.3
Total	100.0	100.0

Looking at the condition of illness (injury) or physical discomfort among the older adult floating population in Shanghai (Table 5), the proportion of men who have not been ill (healthy) in the past year (80.5%) is higher than that of women (76.0%), and the proportion of women who have been ill (injured) or physically uncomfortable within the last 2 weeks (11.5%) and before that (12.5%) is higher than that of men (7.1 and 12.4%, respectively). This fully demonstrates: the self-assessed health level and actual condition of male older adult floating population are both higher than those of women, but the proportion of men who “cannot live independently” is higher than that of women, and the proportions of women who “can live independently” and actual illness rate are both higher than those of men. This may be related to the traditional concept of “male supremacy and female inferiority.” In actual life, female older adult floating population undertake more household chores, and their personalities are relatively gentle, which makes them more likely to feel physically uncomfortable and express their own illnesses (24). Although they often have minor illnesses or chronic diseases, they can still live independently. However, men (especially some young-old who are still working) have greater work pressure, stronger personalities, and are unwilling to express their own emotions. This makes the young-old male migrants who have labor force relatively healthier, while those who have lost their labor force are more likely to suffer from serious illnesses, making it impossible for them to live independently.

**Table 5:** Illness (injury) or physical discomfort by gender.

Illness (injury) or physical discomfort	The proportion of male (%)	The proportion of female (%)
Sick within 2 weeks	7.1	11.5
Sick more than 2 weeks ago	12.4	12.5
No	80.5	76.0
Total	100.0	100.0

##### 3.1.2.2 Divorced older adults have the best health status, followed by first marriage, remarried is worse, widowed is the worst

Shown in Table 6, the health status of widowed migrants is the worst, with the lowest self-assessed health rate (82.2%) and an actual non-illness rate of only 68.9%; the health status of divorced migrants is the best, with the highest self-assessed health rate and actual non-illness rate (100.0% and 87.0% respectively). There is only one unmarried sample among the older adult floating population aged 60 and above, which is excluded from the rankings and detailed analysis.

**Table 6:** Cross-analysis of marital status and the health status of the older adult floating population.

Marital status	The proportion of self-assessed health (%)	The proportion of who actually not sick (%)
First marriage	92.8	80.2
Remarried	92.3	61.5
Divorced	100.0	87.5
Widowed	82.2	68.9

The impact of marital status on the health status of older adult floating population is mainly manifested in: (1) The self-assessed health of widowed older adults is the worst and the actual non-illness rate is very low, which may be related to the loneliness of widowed older adults when they are ill. In the future, more attention should be paid to the mental health education, emotional comfort and psychological care of widowed migrants. (2) The health status of divorced older adults is better, their self-perception of health is the best, and it is relatively close to the actual illness situation. (3) The illness rate of remarried older adults is the highest, but their self-perception of health is much higher than the actual illness situation. This is mainly related to the greater twists and turns of remarried older adults in marriage, the relatively complex relationship among family members, changes in diet structure and lifestyle, etc. In the future, we need to guide remarried older adults to better integrate into remarried families in terms of thoughts, emotions, and life. (4) The health status of first-married older adults is in the middle, and their self-perception of health is higher than the actual illness situation, and they also need to strengthen their understanding of the category of health.

##### 3.1.2.3 Older adult individuals residing in two-person households exhibit the best health status, while those living alone fare the worst

As Table 7 shown, the health status of older adult individuals living alone is the worst, with both their self-assessed health status and actual rate of disease-free status being the lowest. In households with four residents, the proportion of older adult individuals self-rating their health is the highest, yet their actual rate of disease-free status is second to last. Older adult individuals in two-person households have the highest rate of disease-free status, with their self-assessed health status ranking third. It is evident that an excessively high or low number of cohabiting family members is detrimental to the physical health of the older adults in the household. When the number of cohabitants is too low (such as in the case of living alone), the older adult individual may feel overly isolated and lack care when ill, leading to the worst self-perception of health. An excessive number of cohabitants can result in complex family relationships and potential physical and emotional exhaustion. A moderate number of cohabitants, between two and four, can both care for the older adults and facilitate harmonious cohabitation, thus promoting the physical health of the older adults.

**Table 7:** Cross-analysis of the number of cohabiting family members and the health status of the older adult floating population.

The number of cohabiting family members	The proportion of self-assessed health (%)	The proportion of who actually not sick (%)
1	71.4	63.6
2	94.4	82.6
3	95.7	78.7
4	96.6	73.3
5	89.9	74.3
6 and above	87.5	75.0

#### 3.1.3 Self-assessed health and socio-economic factors

##### 3.1.3.1 The health status of older adult floating population with agricultural household registration is better than that of non-agricultural migrants

The self-assessed health status of older adult floating population with agricultural household registration is 92.2%, which is 0.9 percentage points higher than that of non-agricultural migrants. The actual rate of disease-free status is 85.3%, which is 11.3 percentage points higher than that of non-agricultural migrants. This suggests that the older adult non-agricultural migrants in Shanghai are more likely to suffer from chronic diseases such as hypertension and diabetes due to the long-term effects of dietary habits, occupational characteristics, and lifestyle. On the other hand, older adult floating population with agricultural household registration may have better physical fitness due to more physical labor, or they may have a lower use rate of basic public services, leading to missed diagnosis.

##### 3.1.3.2 Individuals with no formal education have the worst self-assessed health, while those with high levels of education have good self-assessed health but a high risk of health issues

According to the self-assessment results (Table 8), among the older adult floating population, those with an associate or bachelor’s degree have the highest proportion of self-assessed health, both at 96.7%; those without formal education have the lowest proportion of self-assessed health, at 78.4%. Specifically, the highest rate of “healthy” self-rating is among those with a bachelor’s degree, at 60%, while the highest rate of “basically healthy” self-rating is among those with an associate degree (54.8%), and the lowest is among those without formal education (27.0%). This suggests that there is a significant positive correlation between the level of education and self-assessed health.

**Table 8:** Cross-analysis of education level and the health status of the older adult floating population.

Education level	The proportion of self-assessed health (%)	The proportion of who actually not sick (%)
Never attended school	78.40	75.70
Elementary school	92.10	85.70
Middle school	95.20	78.80
High school/vocational school	89.60	71.90
Undergraduate	96.70	83.90
Graduate and above	96.70	74.20

From the perspective of disease status, older adult floating population with primary school education have the highest disease-free rate, at 85.7%, followed by those with a bachelor’s degree (about 83.9%), while those with high school/vocational school education and those without formal education have disease-free rates of 71.9 and 75.7% respectively, ranking last.

Compared to the self-assessment results, the self-assessed health level of older adult floating population with different levels of education is higher than their actual health level. Those with an associate or bachelor’s degree have the highest self-assessed health, but their actual disease rate is also high. This may be due to their higher awareness of chronic diseases, but they do not pay enough attention to their impact. Older adults without formal education have the worst self-assessed health, but their actual disease-free rate is not the lowest. This may be due to their dissatisfaction with their health and inaccurate health cognition. Older adult floating population with primary and junior high school education have higher self-assessed health and disease-free rates, which may be related to their relatively low work intensity and slower pace of life. However, it may also be due to their lack of medical awareness and self-care awareness, leading to overestimation of their own health status.

### 3.2 Descriptive results of medical security status of the older adult floating population

The medical security status of the floating population mainly involves issues such as cross-regional medical treatment, participation in medical insurance, and reimbursement.

#### 3.2.1 Hospitalization status

In the 2018 survey of the hospitalization status of the older adult floating population in the past year, only 79 valid questionnaires were obtained. Among them, over 70% of the older adults who were ill were hospitalized in Shanghai, 19.3% were hospitalized in their place of household registration, and 6.9% were hospitalized in other places. This indicates that the cross-regional medical treatment system for the older adult floating population in Shanghai still needs to be improved, with about 30% of the older adults still unable to be treated locally in Shanghai.

#### 3.2.2 Social health insurance

##### 3.2.2.1 Variations in social health insurance participation

In China, the Basic Medical Insurance for Urban and Rural Residents is a policy implemented in certain regions to unify the basic medical insurance coverage for rural residents with local household registration, urban residents with local household registration, and non-local residents. The New Rural Cooperative Medical Insurance is a mutual aid medical insurance system primarily focused on major illnesses for farmers. The Basic Medical Insurance for Urban Residents combines individual contributions with government support and social donations to provide coverage for hospitalization due to major illnesses and special outpatient treatment expenses. Urban Employee Medical Insurance is a form of medical security that involves both the employer and employee, with contributions shared between them, and combines social pooling with individual accounts. The publicly-funded medical care is implemented in China to protect national staff, through which the healthcare departments provide free medical and preventive services to eligible individuals according to regulations. In recent years, with the advancement of the reform of public medical care, relevant personnel will also use medical insurance cards for medical treatment and conduct real-time settlement, similar to basic medical insurance participants.

The highest number of participants in social health insurance is found in the New Rural Cooperative Medical Insurance, accounting for approximately 37.4% of the total. Urban resident medical insurance and urban employee medical insurance both come second, each constituting about 21.3% of the total. The proportion of participants in the basic medical insurance for urban and rural residents is even lower, at 17.7%. The lowest proportion is found in publicly-funded medical care, which only accounts for 2.3%.

##### 3.2.2.2 Reimbursement status of health insurance

###### 3.2.2.2.1 Basic reimbursement situation

The frequency of insurance reimbursements for the older adult floating population in Shanghai is generally low. The highest proportion of reimbursements comes from urban employee medical insurance, accounting for about 41.7%, followed by urban resident medical insurance, accounting for approximately 33.3%. The reimbursement rates for the remaining types of insurance are only about 8.3% each.

###### 3.2.2.2.2 Regional differences in reimbursement

Significant differences exist in the reimbursement of various social health insurance schemes between local areas and places of household registration. In local reimbursements, the highest proportion comes from urban employee medical insurance, at 57.1%, followed by urban resident medical insurance, at approximately 42.9%. Both are higher than their proportions in places of household registration. Publicly-funded medical insurance and basic medical insurance for urban and rural residents have higher proportions in places of household registration (25.0%) than locally (0.0%). The reimbursement rate of the New Rural Cooperative Medical Insurance in both local areas and places of household registration is 0.0%. These findings indicate that the majority of older adult floating population in Shanghai are urban residents (as discussed earlier) and can mostly receive social health insurance reimbursements locally. Among them, working older adult floating population have a higher proportion of urban employee medical insurance reimbursements than non-working older adult floating population. Rural residents are in the minority and must return to their places of household registration for social health insurance reimbursements. The older adult floating population benefits limitedly from social security and welfare, and the reform of social medical insurance in Shanghai and even all over China has a long way to go (39, 40).

###### 3.2.2.2.3 Hospitalization cost expenditure and reimbursement ratio

In general, the reimbursement ratio for older adult floating population in Shanghai is roughly proportional to the cost of hospitalization and medication. In the current sample, based on the exchange rate at the time of writing this article (1 USD = 7.2 Chinese Yuan), the frequency of hospitalization and medication costs between 4,167$ and 12,500$ is the highest (with three people in each category), with individual payments amounting to 2,983$ and 6,944$, respectively, and reimbursement ratios of 50.0 and 66.7%, respectively. The frequency of other hospitalization and medication costs is much lower (only one or zero people). The relationship between individual payments by older adult floating population in Shanghai and hospitalization and medication costs is divided by the boundary of 1,389$. Below 1,389$, individual payments are negatively correlated with hospitalization and medication costs - the lower the hospitalization cost, the higher the individual payment, and the lower the reimbursement ratio. For instance, for an older adult with hospitalization and medication costs of 1,180$, and the reimbursement ratio is 0.0%, meaning that the costs of hospitalization and medication are entirely borne by the individual. However, for an older adult with hospitalization costs of 1,250$, the individual payment is 528$, and the reimbursement ratio is approximately 42.2%. For costs over 1,389$, individual payments are positively correlated with hospitalization and medication costs – the higher the hospitalization cost, the higher the individual payment, but the payment ratio decreases, and the reimbursement ratio increases. For instance, for an older adult with hospitalization and medication costs of 4,861$, the individual payment is 2,778$, and the reimbursement ratio is 57.1%. For an older adult with hospitalization and medication costs of 12,500$, the individual payment is 6,944$, and the reimbursement ratio is approximately 66.7%.

### 3.3 Descriptive results of community health service status of older adult floating population

#### 3.3.1 Status of establishing resident health records

The proportion of older adult floating population who establish health records is relatively low, as is their awareness of the process. From the perspective of local health record establishment, the proportion of those who have already established records is the lowest, at only 9.7%. Among those who have not established health records, 54.9% have never heard of it, 25.6% have heard of it, and about 9.7% of the older adult floating population are unclear about it. This indicates that while the work of establishing health records has a certain degree of awareness among the older adult floating population, various reasons prevent them from undertaking this task, with some lacking understanding. In future work, those who have heard of it but have not established a record should be targeted for promotion and guidance to increase the proportion of health record establishment. For those who have not heard of it and those who are unclear, efforts should be made to increase publicity and education about the establishment of health records and improve their awareness of health record keeping.

#### 3.3.2 Family doctor contracting status

The highest proportion is “heard of it but did not sign,” at 46.3%; the lowest proportion is “signed,” at only 3.0%. The proportions of “never heard of it, never signed” and “unclear” are 43.5 and 7.1%, respectively. This is related to the overall low economic income level of the older adult floating population, their insufficient consumption capacity, relatively traditional thinking, and lack of understanding of the advantages of family doctors. The high proportion of “heard of it but did not sign” indicates that the older adult floating population has a certain basic understanding. In the future, more publicity and education should be carried out in this regard, allowing more older adult floating population to receive better medical services.

#### 3.3.3 Status of receiving community health education

Table 9 shows that the highest proportion of the older adult floating population in Shanghai receives education on chronic disease prevention and control, followed by public event self-rescue, with the lowest proportion receiving occupational disease prevention and control education. Among them, chronic disease prevention and control health education accounts for 24.8% of the total number of people receiving health education, reflecting the effectiveness of the country’s promotion and management of chronic disease prevention and control. The proportion of occupational disease prevention and control education is the lowest, accounting for only 9.4% of health education. The health consciousness of the migrant population is relatively weak, and they often do not consider occupational disease factors when faced with health problems. Increasing occupational disease health education for the older adult floating population can enhance their protective awareness at work, thereby effectively improving the health level of the older adult population.

**Table 9:** The proportion of various disease education in health education.

Types of health education	Proportion (%)
Occupational disease prevention	9.40
Infectious disease prevention	14.80
Reproductive health and maternal and child health	12.50
Chronic disease prevention	24.80
Mental health (including mental disorder prevention)	12.50
Self-rescue in emergency public events	15.70
Others	10.30

## 4 Main factors affecting the health of the older adult floating population

### 4.1 Model and indicator selection

The primary factors affecting the health of the older adult floating population are analyzed by using the binary Logistic regression model constructed with formulas (1, 2). The dependent variables “self-assessed health status” and “actual disease status” are both binary variables, and the independent variables are all categorical variables. Firstly, individual factors such as the gender, age, educational level, and marital status of the older adult floating population are selected as control variables (Model 1). Based on this, social-economic factors such as household registration, family monthly income, housing expenditure (Model 2), migration factors such as place of origin, length of stay in Shanghai, reasons for migration, willingness to stay (Model 3), and medical factors such as the status and method of receiving community health education, participation in medical insurance (Model 4) are included. Finally, all types of factors are included (Model 5) for comprehensive consideration. The model results are detailed in Table 6.

### 4.2 Results analysis

As shown in Table 10, in Model 1, the health status of the older adult floating population is significantly influenced by age and educational level, while gender and marital status do not have a significant impact. In terms of age, the health status of older adult migrant population aged 60–64 is the best, while that of those aged 75–79 is the worst. However, for those over 80, the risk of illness slightly decreases, and health status slightly improves. The older adults in this age group have a lower resistance and recovery ability to diseases, which reduces the proportion of sick individuals to a certain extent. This could also be related to changes in lifestyle and the use of medical services among the older adults as they age. As they grow older, they may pay more attention to healthy eating habits, regular check-ups, and more frequent use of medical services, which could contribute to improving their health status and reducing the chance of illness. In terms of education, compared to the older adult floating population with primary school or lower education, those with junior high school, high school, and college or higher education have a 3.49, 1.61, and 4.90 times higher probability of self-assessed health respectively, indicating a positive correlation between educational level and the health status of the older adult floating population. This could be because the older adult floating population with a higher level of education are more capable of obtaining, understanding, and utilizing health-related information. They may have a better understanding of healthy lifestyles, such as balanced diet, moderate exercise, as well as methods for preventing and managing chronic diseases. Simultaneously, older adult individuals with higher education usually have higher incomes, a higher standard of living, and a greater ability to bear healthcare costs. Moreover, highly educated individuals often have broader social networks, which could provide more social support when facing life’s pressures and challenges, benefiting their physical and mental health.

**Table 10:** The main influencing factors of the health status of the older adult migratory population in the binary logistic regression model.

Variables	Model 1		Model 2		Model 3		Model 4		Model 5	
	Self-assessed	Disease conditions	Self-assessed	Disease conditions	Self-assessed	Disease conditions	Self-assessed	Disease conditions	Self-assessed	Disease conditions
Individual factors										
Male	0.841	0.721	0.917	0.751	0.486	0.859	0.730	0.814	0.448	0.998
Ages										
65–69 years old	0.242^**^	1.876^*^	0.244^**^	1.739	0.256^**^	1.773	0.263^**^	1.925^*^	0.261^*^	1.772
70–74 years old	0.399	1.137	0.617	0.914	0.601	0.886	0.352	0.986	1.004	0.641
75–79 years old	0.069^***^	4.843^**^	0.092^**^	3.785^*^	0.128^*^	2.644	0.064^***^	5.565^**^	0.152^*^	2.527
80 years old and above	0.199	3.104	0.184	2.689	0.366	2.516	0.210	3.159	0.372	2.298
Education level										
Middle school	3.485^*^	1.242	4.684^*^	0.978	5.269^**^	1.025	4.602*	1.229	12.607^**^	0.792
High school/Vocational school	1.610	1.735	2.113	1.214	1.822	1.763	1.362	1.618	2.026	1.106
Undergraduate and above	4.904^*^	1.112	6.646^*^	0.728	6.473^*^	0.945	3.788	1.437	8.526^*^	0.742
Marital status										
With a spouse	1.150	0.889	1.039	0.839	1.714	0.729	0.986	0.939	1.025	0.768
Socio-economic factors										
Household registration										
Non-agricultural household registration			1.592	0.590					1.205	0.468
Household monthly income										
Medium-income			2.603	0.872					3.074	0.825
High-income			1.243	1.074					1.304	0.950
Housing expenditure										
Medium			3.455	0.450					3.224	0.511
High			0.459	1.438					0.262	1.590
Migration factors										
Place of household registration										
Central					1.613	1.759			2.301	1.750
West					0.853	1.225			1.504	1.096
Northeast					1.891	1.229			2.410	1.026
Length of stay in Shanghai										
5–9 years					1.182	0.909			1.444	1.025
10–14 years					0.598	1.740			0.853	1.549
15–19 years					0.223^*^	3.270^*^			0.169^*^	3.001^*^
20 years and above					0.289	1.095			0.278	1.090
Reason for migration										
Family migration					0.314	1.365			0.345	1.013
Visiting friends and relatives					0.503	0.895			0.183	1.206
Aging in a different place					1.573	0.744			0.807	0.692
Willingness to settle										
Yes					1.971	1.099			1.950	1.212
Healthcare factors										
Receive health education										
Yes							2.030	0.572	1.660	0.630
Medical insurance										
Yes							0.770	1.396	1.072	1.461

^*^represents *p* < 0.05, ^**^represents *p* < 0.01, ^***^represents *p* < 0.001.

The results of Model 2 indicate that household registration does not significantly affect the self-assessed health and disease conditions of the older adult floating population. Family monthly income only has a slight impact on self-assessed health, while housing expenditure only slightly affects the disease conditions. This suggests that household registration does not cause significant differences in economic conditions and medical resources among the older adult floating population. In terms of income level, the self-assessed health of older adult floating population with moderate household income is better than that of those with lower household income (*p* = 0.093), while the self-assessed health of older adult migrant population with low household income is relatively low. In terms of housing expenditure, the disease risk of older adult floating population with medium housing expenditure is lower than that of older adult floating population with lower housing expenditure (*p* = 0.089), suggesting that a better living environment can reduce the disease risk of the older adult floating population. Specifically, housing conditions (such as ventilation, lighting, and heating systems), living environment (such as air quality), and community services (such as fitness facilities) may all influence the health status of the migrant older adult population. In general, low family income and poor housing conditions are the primary economic factors affecting the health status of the older adult floating population. The medical burden caused by illness also affects family economic conditions and housing affordability, leading to a poverty trap.

The results of Model 3 indicate that the region of migration only has an impact on the risk of illness among older adult floating populations, while the reason for migration only affects the self-assessed health. The duration of residence in Shanghai has an impact on both s self-assessed health and illness status, while the willingness to reside has no significant influence on either of them. From the perspective of the migration region, the disease risk of older adult floating population from the central region is 75.9% higher than that of older adult floating population from the eastern region (*p* = 0.082). This could be due to the lower quality of life and fewer medical resources in the central region, leading to a decline in the physical fitness of the older adult floating population. On the other hand, it could be because the older adult floating population from the central region are not accustomed to the environmental differences after moving to Shanghai. In terms of duration of stay in Shanghai, the self-assessed health results of older adult floating population who have stayed in Shanghai for more than 10 years are worse, and the disease risk increases. The duration of stay in Shanghai is related to the age of the migrant older adult and reflects the long-term negative impact of living in Shanghai on the physical health of the older adult floating population. This could be related to the high population density in Shanghai and the overcrowding of public resources such as medical care and green spaces, which are not suitable for older adult residents. The research also found that the self-assessed health results of older adult floating population who moved for family reasons are worse (*p* = 0.086), which might be related to their difficulty in adapting to the climate, neighborhood atmosphere, and lifestyle of big cities (41). The intention to stay does not significantly affect the self-assessed health and disease conditions of the older adult floating population, indicating that the intention to stay does not affect their health status and quality of life.

The results of Model 4 show that the probability of self-assessed health among older adult floating population who have received community or workplace health education is twice as high as that of those who have not (*p* = 0.096), and their disease risk is 42.8% lower (*p* = 0.055). This suggests that community/workplace health education can enhance the awareness of medical care among the older adult floating population, increase their utilization probability of health service resources, and further improving their health status. Participation in medical insurance does not significantly affect the self-assessed health and disease conditions of the older adult floating population. This could be because the uninsured sample individuals are generally healthier and do not need insurance, or it could be related to the high requirements of insurance for individual age and health status, resulting in a smaller sample size of insured migrant older adult individuals.

After incorporating all variables into Model 5, the impact of individual factors such as age and educational level on the health of the older adult floating population significantly increased. Among socio-economic factors, higher housing expenditure has a negative impact on self-assessed health results (*p* = 0.073). Among migration factors, the duration of stay in Shanghai still has a long-term negative impact on the older adult floating population. Among medical factors, the impact of whether to receive medical education on the health of the older adult floating population has become insignificant, with age and educational level remaining as the main factors affecting the health and disease risk of the older adult floating population.

Overall, older adult individuals of advanced age and lower educational level are at health risk. A better living environment can reduce the disease risk of the older adult floating population. Low family income, poor ability to pay for housing, and the medical burden caused by illness can lead to a poverty trap for the older adult floating population. Older adult individuals from the central region and those who migrated for family reasons may have difficulty adapting to life in big cities, resulting in poorer self-assessed health. These situations require serious attention from relevant government departments. Meanwhile, community/workplace health education can enhance the health protection awareness of the older adult floating population. In the future, efforts can be made to further increase health education promotion and encourage more older adult individuals to learn more about health knowledge.

## 5 Conclusion and policy implication

### 5.1 Conclusion and discussion

This study analyzes the demographic characteristics and health status of the older adult floating population in Shanghai in the new era, and reveals its main influencing factors. It was found that: (1) Over 90% of the older adult floating population self-rated their health (with only a very small number of older adults unable to care for themselves), which is much higher than the proportion of older adults who are not sick (injured) or physically uncomfortable (i.e., actually healthy) which is over 70%. (2) The health status of the older adult floating population deteriorates with age, and those who have never attended school and live alone have the worst health. The older adult floating population lack health knowledge and have a low proportion of local reimbursement. Widowed and low-educated older adult floating population urgently need attention. (3) The older adult floating population with advanced age and low education level are at health risk. A better living environment can reduce the risk of illness in the older adult migrant population. Lower family income, poor housing payment ability, and the medical burden brought by illness can easily lead to the older adult floating population falling into the poverty trap in old age. The older adult floating population from central regions and migrated older adults have difficulty adapting to life in big cities, which leads to poor self-rated health. Meanwhile, community/unit health education is beneficial to improve the health protection awareness of the older adult floating population.

This article still has some issues need discussion. Firstly, in this study, the probabilities of self-rated health among the older adult floating population with junior high school, high school, and college or higher education are 3.49 times, 1.61 times, and 4.90 times that of those with primary school or lower education, respectively, far higher than the probability multiples of 1.68, 1.79, and 1.61 in another study. The health status differences between the older adults with different levels of education, compared to non-migrants, may result in greater differentiation, which calls for further research (42). Secondly, although some studies have revealed that marriage helps maintain health by protecting people from physical and emotional stress and harmful health behaviors (43, 44), this study did not find a statistically significant difference in the health status of older adult migrants with or without spouses. Some research has pointed out during analysis that compared to rural areas with closer social relations, older adults in Indian cities mostly live in nuclear families, and their health status is more affected by marital status (45, 46). Similarly, it can be speculated that although the reasons for migration vary among the older adult floating population in this study, the act of migration itself implies exposure to new social networks, which might dilute the impact of marital status on health status to some extent. Thirdly, the relationship between family income and older adult health is complex, and the self-assessed health and actual disease conditions of the older adults may also be influenced by various factors such as family atmosphere and relationships among family members, which require further research. Fourthly, although this study did not directly compare and analyze the health status of older adult floating populations with that of ordinary older adults, by comparing the self-assessed health levels of the older adults in this study with those in other research, it was found that the self-assessed health of older adult migrant populations in Shanghai is better than the average level of older adults in China, indirectly indicating that the theory of healthy selection can be used to a certain extent to guide research on older adult floating populations in China (47).

Overall, this research can to some extent reflect the characteristics and health status of the older adult floating population in Shanghai in the new era, and reveal the main factors affecting the health of the older adult floating population in Shanghai. The corresponding countermeasures and suggestions proposed in this study can provide empirical evidence for the next step in advancing the policy formulation for the older adult floating population. The research has significant practical implications for enhancing the attention to the policies of the older adult floating population, promoting the equalization of public and health services, and responding to the implementation of the proactive strategy to cope with aging at the national level.

This study has some limitations too. Firstly, although this study uses the latest batch of data published by CMDS, the survey was conducted in 2018, which may not fully reflect the current situation. As time goes by, the size, structure, and health status of the older adult floating population may change. Secondly, due to data limitations, this study did not explore factors such as social support and cultural adaptation for the older adult floating population. Thirdly, due to the cross-sectional nature of the data, this paper can only engage in a discussion about correlational relationships. Whether there is a causal relationship between influencing factors and health status, as well as specific mechanisms, require further in-depth research and discussion.

### 5.2 Policy implications

#### 5.2.1 Strengthening the management of the inflow of the older adult migratory population and enhancing integrated supervision, optimizing top-level questionnaire design, and improving the construction of information sharing platforms

The presence and continuous growth of the older adult migratory population in China’s major cities, notably Shanghai, is a clear and long-term trend. Government departments should regard this phenomenon as the foundation for the establishment of relevant laws, policies, and systems. The establishment of an internet monitoring system and big data management platform for the older adult migratory population should be prioritized, along with an enhancement of spatial integrated supervision. Policies should ideally lean toward attracting younger, highly educated older adult individuals, thus guiding the balanced development of Shanghai’s older adult migratory population. For instance, household registration behaviors of the older adults are selective; compared to those with agricultural identities, non-agricultural older adult individuals are more likely to join the influx into Shanghai.

Simultaneously, departments such as the National Health Commission should further improve data collection work for the older adult migratory population, optimize top-level questionnaire design, particularly in the health sector, and draw from comprehensive international health evaluation indicators to establish a health monitoring mechanism for the older adult migratory population. This mechanism should include exhaustive objective evaluation questions to assess the daily living self-care ability, frailty, and chronic conditions such as hypertension and diabetes among the older adult migratory population. It should also encompass comprehensive surveys on the psychological health, intergenerational family relationships, and social integration of the older adult migratory population.

In addition, the migratory population information management system should be further optimized. Collaboration with higher education population research institutions and experts should be enhanced, aiming to achieve data sharing, integration, and expansion, reduce repeated data entry, and facilitate the conversion of scientific research outcomes. This will provide a scientific basis for government departments to improve social pension, medical insurance, and other policies.

#### 5.2.2 Paying attention to the health status of the older adult with high age and low education, promoting older adult education, and focusing on vulnerable groups such as widowed older adults

The health status is a prerequisite for the mobility of the older adults. The health status of older adult individuals is generally poor, and those with lower education levels lack correct understanding of the relationship between disease types and health status. With increasing age, the rates of self-assessed health and non-illness decline, while the rates of self-assessed unhealthiness and illness rise, indicating that the health status of older adult individuals is poorer. At the same time, there is a clear positive correlation between the level of education and self-assessed health. Older adult migratory individuals who have never attended school have the lowest rate of illness but also the lowest rate of self-assessed health, indicating a lack of correct understanding of the relationship between disease types and health status. The self-assessed health of the older adult migratory population in Shanghai tends to be overly optimistic, with self-perceptions of good physical health often higher than actual illness conditions, and perceptions of poor health far lower than actual illness rates. This is related to the lack of medical knowledge among the older adult migratory population, who often misinterpret some chronic diseases as normal phenomena of aging and self-rate as healthy or basically healthy.

Therefore, relevant departments should strengthen the propaganda of chronic disease prevention and treatment, first aid knowledge, and health preservation knowledge for the older adults, enhance older adults’ objective understanding of their own physical health status, and their ability to resist “the risks of longevity.” Increased investment in health training in public services and communities/units should be made, making full use of health service resources to improve the health care awareness of the older adult migratory population.

Secondly, government departments such as the Health Commission and the Civil Affairs Department, especially community older adult care service institutions, should pay special attention to widowed and low-educated migratory populations, groups with prominent health risks. They should provide care and policy inclination in health checkups, medical care, community services, home-based older adult care, and government-purchased older adult services to ensure they enjoy relatively fair public social services, safeguard their basic social rights and interests, and enhance the quality of health services for the older adult migratory population.

In addition, given that the rate of widowhood among the older adults with a higher level of education is significantly lower than that of the older adults with a lower level of education, the government and relevant social departments should increase their focus and promote older adult education. This includes education and guidance on psychological health knowledge, early childhood education knowledge, medical first aid knowledge, health preservation knowledge, anti-fraud knowledge, and education on interest-related knowledge such as entertainment, tourism, and finance. This will enhance the cultural level of the older adults, and strengthen their self-protection and health protection capabilities.

Psychological counseling on dealing with the emotional comfort of widowed and retired older adults, the feeling of loneliness from not being able to integrate into city life, and the expansion of interest hobbies should be provided to enrich the spiritual world and quality of life in their later years. This ensures that they can have a healthy and happy later life. Special attention should be paid to the daily life, material, and spiritual care of widowed older adults (especially low-educated female widowed older adults). Efforts should be made to improve their educational level, improve their ideas and methods of caring for grandchildren, enhance their ability to handle family conflicts, alleviate generational conflicts, and enhance their ability to integrate into society.

#### 5.2.3 Accelerating the advancement of the inter-provincial offsite medical insurance reimbursement mechanism, promoting the integrated development of older adult care in the Yangtze River Delta

The health status of Shanghai’s migratory older adult population is good, but there are significant differences in the types and locations of social medical insurance participation, and the local reimbursement frequency of various insurances is generally low. With the extension of expected life span, degenerative diseases are gradually becoming potential health risks for the older adult migratory population. However, the insurance status of the older adult migratory population is not optimistic. There are significant differences in the places of participation in social medical insurance, and the proportion of participation in various types of insurance is mainly based on the place of household registration. The reimbursement frequency of all types of insurance is generally low, and the proportion of insurance reimbursements in the local area is much lower than that in the place of household registration (except for urban employee medical insurance). Non-agricultural working migratory older adults who come to Shanghai can mostly carry out social medical insurance reimbursement locally. However, those who are jobless and those with agricultural household registration cannot get medical reimbursement locally and have to return to their place of household registration for social medical insurance reimbursement. It is evident that there is still a long way to go in health knowledge propaganda education and reform of the social medical security system in major cities represented by Shanghai and even nationwide.

Therefore, against the backdrop of the Yangtze River Delta integration rising to a national strategy, government departments at all levels in Shanghai should actively respond to the call of General Secretary Xi Jinping and the Party Central Committee, make the promotion of the integrated construction of older adult care in the Yangtze River Delta a key work of relevant departments in Shanghai, Zhejiang, Jiangsu, and Anhui. Based on the basic reality of regional governance at home and abroad, adhere to the key concept that the key to social governance is the governance of mobility, public relations have mobility, optimize social structure, rationally allocate resources spatially, respect individual mobility decisions and regulate their behaviors, establish the Yangtze River Delta Older adult/adults Association consortium. With the pilot cities as the focus, carry out mutual recognition and research on older adult care needs assessment, older adult service-related standards, and the evaluation system of the older adult care team, establish a mechanism for mutual recognition and promotion of high-quality and trustworthy older adult service brands, and other tasks. The government should formulate policy plans, vigorously strengthen the integrated construction of social older adult care security for the older adult migratory population in the Yangtze River Delta, rationally plan the layout of the Yangtze River Delta older adult service industry, accelerate the construction of integrated medical insurance in the Yangtze River Delta, strengthen inter-provincial offsite medical insurance cooperation, establish a management platform for offsite medical treatment personnel, medical treatment and settlement of accounts, and solve the worries of offsite medical treatment and health care for urban older adult migratory populations.

## Data availability statement

The original contributions presented in the study are included in the article/supplementary material, further inquiries can be directed to the corresponding author.

## Ethics statement

The studies involving humans were approved by Shanghai Municipal Health Commission. The studies were conducted in accordance with the local legislation and institutional requirements. The participants provided their written informed consent to participate in this study.

## Author contributions

LW: Conceptualization, Formal analysis, Funding acquisition, Writing – original draft, Writing – review & editing, Data curation, Project administration. WL: Formal analysis, Data curation, Writing – review & editing. SW: Data curation, Software, Methodology, Writing – review & editing. GW: Funding acquisition, Supervision, Writing – review & editing. XW: Data curation, Methodology, Writing – review & editing.
